# Genetic variation among mainland and island populations of a native perennial grass used in restoration

**DOI:** 10.1093/aobpla/plt055

**Published:** 2013-12-18

**Authors:** Kristina M. Hufford, Susan J. Mazer, Scott A. Hodges

**Affiliations:** 1 Ecology, Evolution and Marine Biology, University of California, Santa Barbara, CA, USA; 2 Present address: Ecosystem Science and Management, University of Wyoming, Laramie, WY, USA

**Keywords:** AFLP markers, California Channel Islands, ecological restoration, *Elymus glaucus*, genetic drift, seed source, self-pollination, spatial genetic structure.

## Abstract

Genetic marker studies can assist restoration practice through selection of seed sources that conserve historical levels of gene diversity and population genetic differentiation. We examined genetic variation and structure within and among mainland and island populations of *Elymus glaucus*, a perennial bunchgrass species native to western North American grasslands that is targeted for grassland restoration. Island populations of *E. glaucus* represent sensitive sites and potentially distinctive seed sources for reintroduction, and little is known of their genetic composition. Genetic diversity and structure were estimated using amplified fragment length polymorphism markers for 21 populations and 416 individuals distributed across two coastal California mainland locations and three California Channel Islands. Eight primer combinations resulted in 166 markers, of which 165 (99.4 %) were polymorphic. The number of polymorphic bands was significantly greater among mainland populations relative to island sites, and locally common alleles were present for each sampled island and mainland location. Population structure was high (62.9 %), with most variation (55.8 %) distributed among populations, 7.1 % between mainland and island locations, and the remainder (37.1 %) within populations. Isolation by distance was only apparent among islands. Using marker data to recommend appropriate seed sources for restoration, *E. glaucus* seeds are best derived within islands with collections representing a large number of individuals from matching environments. Given the limited gene flow and prior evidence of adaptive divergence among populations of this species, regional collections are recommended in all cases to maintain diversity and to avoid long-distance introductions of highly differentiated plant material.

## Abstract (precis)

Knowledge of species-level patterns of genetic diversity can inform and improve protocols when population reintroduction is a restoration objective. We describe the population genetic structure of a geographically widespread species, *Elymus glaucus*, which is now rare in temperate grasslands as a result of biological invasion and land conversion. Our study contrasts data for mainland and Channel Island locations, and makes recommendations for seed provenance selection in ecological restoration using genetic marker data and considering prior field studies of adaptive divergence

## Introduction

Widespread anthropogenic disturbance and introductions of invasive species have resulted in the fragmentation and conversion of grassland ecosystems worldwide (D'Antonio and Vitousek 1992). Temperate grasslands originally dominated by caespitose (bunchgrass) species have been altered by introduced livestock, and are highly vulnerable to invasion by competitive annual and rhizomatous perennial exotic grasses (Mack 1989; Hayes and Holl 2003). California grasslands represent an ecosystem where plant community conversion is nearly complete (Mensing 1998). Mediterranean annual grasses and forbs introduced over the past three centuries now dominate the landscape, and native species exist as remnant populations in a matrix of exotics. Efforts are ongoing to control invasive species and to re-establish native perennial bunchgrasses (e.g. Moyes *et al.* 2005; DiTomaso *et al.* 2007; Cox and Allen 2011), and guidelines are needed for the restoration of sustainable and diverse plant populations.

Fragmented populations of plant species are susceptible to environmental, demographic and genetic stochasticity (Shaffer 1981; Lande 1988). Restoration programmes commonly mitigate environmental and demographic concerns for all target species by increasing the number of both individuals and populations to minimize the probability of extirpation. In contrast, attempts to mitigate the loss of genetic variation and to minimize inbreeding have largely focused on rare and endangered species (Lande 1988), although interest in the genetic consequences of establishing or restoring populations of common species has grown over the last decade (e.g. Hufford and Mazer 2003; McKay *et al.* 2005; Bischoff *et al.* 2010). Evidence suggests that common species are subject to genetic erosion resulting from habitat fragmentation at similar or even greater rates than rare species (Honnay and Jacquemyn 2007). Genetic variation is the basis of adaptation, and the loss of genetic diversity, particularly for fitness-related traits, will impact population persistence as well as limit the ability of a population to adapt to changing environments (Frankham *et al.* 2002; Reed and Frankham 2003). Knowledge of species-level patterns of genetic diversity can inform and improve restoration protocols when population reintroduction is a restoration objective.

Primary genetic concerns for reintroduction include the maintenance of patterns of diversity within and among populations, and the introduction of genotypes adapted to environmental conditions at the restoration site (e.g. McKay *et al.* 2005). Population genetic structure is a function of a species' mating system and provides an estimate of historical levels of gene flow and connectivity among locations (Slatkin 1987). Data that provide measures of the partitioning of genetic variation, however, do not explain underlying causes of divergence, which may be a function of selection or random processes (Heywood 1991). Direct evaluations of genotypic adaptation and traits under selection can be determined in common garden and reciprocal transplant studies (Linhart and Grant 1996; Kawecki and Ebert 2004). When available, these studies provide valuable information for the identification of appropriate seed sources for reintroduction. However, data for common gardens are not available for all species, are limited in scale and may not detect all components of local adaptation (Nuismer and Gandon 2008). Thus, baseline information to describe patterns of genetic variation within and among populations remains relevant for restoration and conservation planning, and can serve as a first step for management of genetic diversity in species reintroduction or augmentation programmes.

The genetic consequences of seed introduction during restoration may have greater impacts for populations occupying small or isolated islands relative to mainland sites. Island populations often harbour lower levels of gene diversity and higher levels of differentiation when compared with the mainland, and are at increased risk of extinction—possibly due to greater environmental and demographic stochasticity (Frankham 1997). Islands are also disproportionately vulnerable to biological invasion, and introduced species are reported to outnumber native species in grasslands of the California Channel Islands (Halvorson 1992; Schoenherr *et al.* 1999; Moody 2000). *Elymus glaucus* (blue wildrye) is a native bunchgrass species once common in California mainland and island grasslands (Holstein 2001; Bartolome *et al.* 2004). Over the past few decades, *E. glaucus* has been a target of restoration programmes due to its wide distribution, wildlife habitat value and dense root system, which prevents erosion in degraded landscapes (Knapp and Rice 1996; Erickson *et al.* 2004). Two previous reciprocal transplant studies found evidence of ecotypic variation among populations of this species as a result of adaptation to local environments over scales of 50–190 km (Hufford *et al.* 2008; Knapp and Rice 2011). These studies were conducted at mainland locations, and no information is yet available to describe genetic differentiation or adaptive variation for populations on the Channel Islands.

In this study, we used amplified fragment length polymorphisms (AFLPs) to characterize genetic diversity and structure among mainland and island populations of *E. glaucus* to address the following questions relevant for grassland restoration: (i) How much genetic variation is present in island populations, and how does this compare to mainland populations? (ii) Is genetic differentiation between island populations greater relative to nearby mainland locations? (iii) Is genetic distance correlated with geographic distance within and among islands and the mainland? Lower levels of diversity and strong genetic differentiation at island sites may indicate the reduced ability of island plants to adapt to altered environments and a greater risk of local extinction of Channel Island populations. At the same time, the geographic scale of genetic differentiation serves as an indicator of the historical rates of gene flow. These data can assist with seed provenance selection for the restoration of *E. glaucus* growing in California coastal and island grasslands by means of maintaining population genetic diversity and lowering the risk of introducing maladapted genotypes.

## Methods

### Study species

The genus *Elymus* includes 150 species distributed in temperate regions worldwide (Hickman 1993). *Elymus glaucus* is a perennial, non-rhizomatous bunchgrass with a broad geographic distribution from Canada to Mexico, and can be found throughout the western United States (Hickman 1993). Herbarium records for this species include much of the state of California (http://www.calflora.org), but extant populations are highly fragmented as a result of widespread land development and biological invasion (Barry *et al.* 2006). Populations of *E. glaucus* occur in diverse habitats and plant communities, and exhibit morphological variation across their range (Snyder 1951; Wilson *et al.* 2001). Polyploidy is common in the Poaceae, and *E. glaucus* is an allotetraploid (2*n* = 28) derived from *Hordeum* and *Pseudoroegneria* ancestors (Jensen *et al.* 1990). Previous studies of *E. glaucus* suggest that it is frequently self-pollinated and has a mixed mating system, allowing for some outcrossed pollination (Knapp and Rice 1996; Ie 2000; Wilson *et al.* 2001). Inflorescences are distinctive, narrow spikes, and seed dispersal is typically passive.

### Study sites and collections

**Figure 1.**
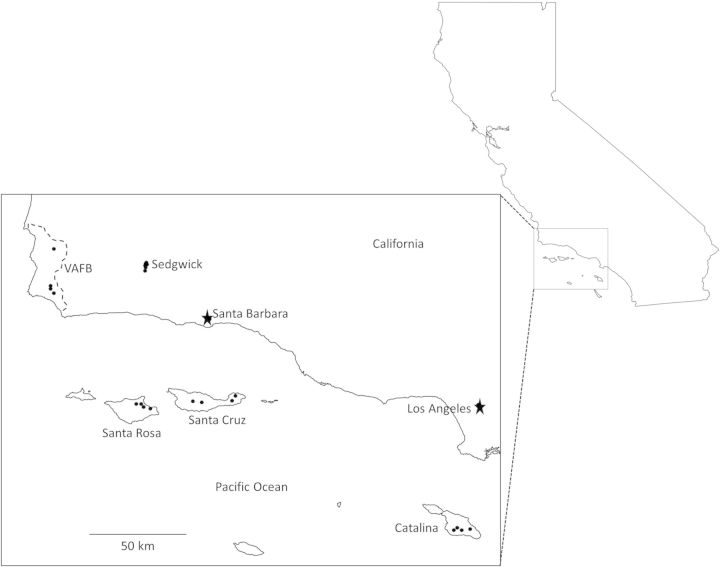
Map of *E. glaucus* study sites in Southern California among the three California Channel Islands and two mainland locations.

The Southern Channel Islands represent an archipelago of eight continental islands located at distances ranging from 13 to 61 km off the coast of mainland California (Schoenherr *et al.* 1999). Despite their proximity to the continental landmass, high numbers of endemic species (up to 47 % of native vegetation) are found in the island chain (Junak *et al.* 1995; Moody 2000). Introduced plants and animals threaten native species, and efforts to conserve and restore island ecosystems are ongoing (Halvorson 1994). In the present study, sampling sites included 21 populations distributed among two mainland locations and the three Channel Islands where *E. glaucus* is known to occur (Junak *et al.* 1997). The mainland sites were located at the University of California Sedgwick Reserve (Sedgwick) in Santa Ynez, California and Vandenberg Air Force Base (VAFB) in Lompoc, California. Offshore sites were located on Santa Rosa and Santa Cruz Islands in Channel Islands National Park, and Santa Catalina Island (Fig. 1).

**Figure 2.**
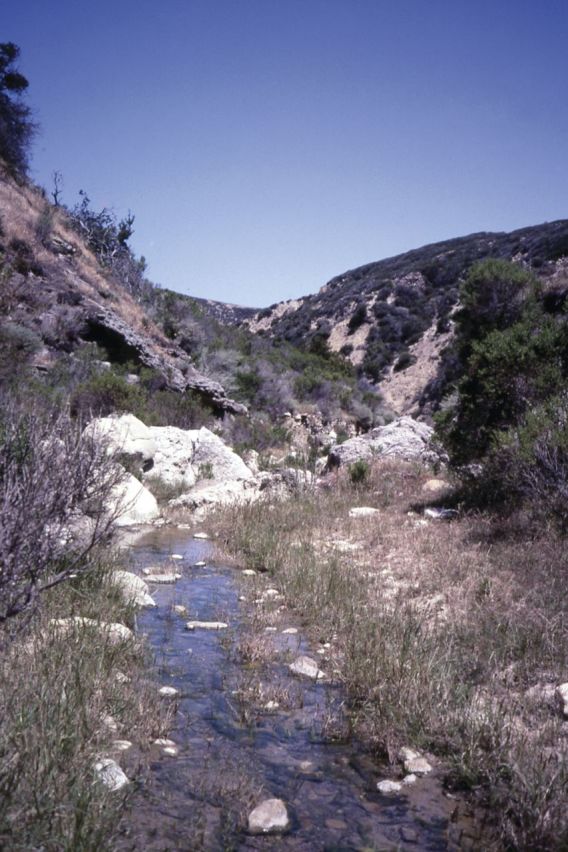
Riparian site representing potential *E. glaucus* habitat on Santa Rosa Island.

Sampling locations represented diverse habitats and serpentine rock outcrops are common at the two mainland locations and Santa Catalina Island. Populations of *E. glaucus* sampled in this study occurred in oak woodland savannahs at Sedgwick and in open, coastal grasslands at VAFB. Island populations were distributed among coastal prairies and ephemeral, riparian environments (Fig. 2). The regional climate is Mediterranean, but due to the marine influence, coastal areas of VAFB and the Channel Islands experience cooler temperatures and higher humidity when compared with the interior of Santa Cruz Island and Sedgwick Ranch. Sites located at Sedgwick and in Santa Cruz Island's Central Valley are subject to greater temperature fluctuations than coastal areas, and often record seasonal temperature differences of 5 °C or more relative to the shoreline (Schoenherr *et al.* 1999).

Populations of *E. glaucus* were identified and georeferenced during several trips to each location in the spring and summer of 2002 and 2003 (Table 1). At each location, we sampled populations that represented geographically distant sites although some areas were inaccessible, limiting our sampling range. Efforts were made to sample plants separated by 0.5 m or greater within populations along 10- to 20-m walking transects. The patchy nature and small size of many *E. glaucus* populations, however, restricted the area within which we were able to collect leaf material, and in some cases we modified transect sampling to collect in a smaller radius while maintaining the separation of sampled plants. In general, population sizes ranged between 20 and 60 visible plants. At each site, leaves from 20 individuals were collected and stored in sealed bags containing silica gel for preservation. One population located on Santa Cruz Island was very small, and only allowed for 16 individual collections.

**Table 1.** Sampled locations and genetic diversity indices for 21 *E. glaucus* populations including latitude (N°) and longitude (W°), sample size (*n*), number of locally common (*f*) and fixed bands (FB), per cent polymorphic bands (PPB), per cent polymorphic loci (PLP) and expected heterozygosity (*H*_e_) with standard errors

										*H*_e_	
Location	N°	W°	Description	Population ID	*n*	ƒ	FB	PPB (%)	PLP (%)	Mean	SE
Santa Catalina	33 20.81	118 26.65	Bullrush Canyon	C1	20	16	75	32.7	30.9	0.120	0.015
	33 21.43	118 25.57	Cape Canyon	C2	20	15	59	50.9	50.6	0.118	0.012
	33 20.85	118 24.32	Middle Canyon	C3	20	5	31	72.7	72.3	0.213	0.015
	33 21.19	118 21.63	Haypress	C4	20	13	40	60.6	56.0	0.157	0.013
Santa Cruz	34 00.75	119 47.77	Portezuela	SC1	20	8	82	25.5	25.3	0.095	0.014
	34 00.35	119 44.96	Valley Road	SC2	20	4	97	18.8	18.7	0.075	0.013
	34 00.94	119 35.55	End of the Line	SC3	20	2	77	28.5	28.3	0.101	0.014
	34 02.44	119 34.45	Scorpion Canyon	SC4	16	8	100	5.5	4.8	0.013	0.005
Santa Rosa	33 59.82	120 05.25	Lobo Canyon	SR1	20	4	80	23.6	22.4	0.053	0.009
	33 59.87	120 03.70	Cherry Canyon	SR2	20	3	77	30.9	30.7	0.069	0.010
	33 58.97	120 02.98	Water Canyon	SR3	20	5	58	47.3	47.0	0.117	0.013
	33 58.55	120 01.00	Box Canyon	SR4	20	7	38	61.2	59.8	0.150	0.013
Sedgwick	34 43.32	120 02.16	Figueroa 4	S1	20	4	79	23.0	22.4	0.060	0.011
	34 43.24	120 02.18	Figueroa 3	S2	20	8	55	49.1	48.8	0.089	0.009
	34 43.00	120 02.34	Figueroa 2	S3	20	6	52	51.5	51.2	0.141	0.013
	34 42.62	120 02.40	Figueroa 1	S4	20	4	97	18.2	18.1	0.035	0.008
	34 41.43	120 02.75	Ranch House	S5	20	13	36	67.3	66.9	0.211	0.015
VAFB	34 48.08	120 30.86	Campground	V1	20	8	61	47.3	47.0	0.143	0.015
	34 36.61	120 31.90	Pasture	V2	20	10	62	50.3	50.0	0.123	0.012
	34 35.64	120 31.91	San Miguelito	V3	20	15	31	65.5	65.1	0.141	0.012
	34 34.30	120 30.92	Sudden/Ave I	V4	20	13	87	24.8	24.7	0.070	0.011
				Island	236		67.8	98.8	37.2	0.107	0.015
				Mainland	180		62.2	96.4	43.8	0.113	0.018
				Mean				40.7	40.0	0.109	0.011

### AFLP genotyping

Leaves stored in silica gel were transported to the University of California in Santa Barbara and stored at an average room temperature of 20 °C prior to AFLP genotyping. For each sampled plant, total genomic DNA was extracted from ∼20 mg of silica-dried leaf tissue using the DNeasy Plant Mini Kit (Qiagen). A total of 416 plants representing 21 *E. glaucus* populations distributed among 9 mainland and 12 island sites were included in subsequent marker analyses.

Molecular markers were amplified following the protocol of Vos *et al.* (1995) with little modification. Approximately 250 ng of DNA were digested with EcoRI and MseI restriction enzymes and ligated to corresponding adapters. The restriction–ligation mix was diluted 1 : 10 and polymerase chain reaction (PCR)-amplified with EcoRI-A and MseI-C pre-selective primers. The resulting PCR template was diluted 1 : 10 prior to amplification with selective EcoRI primers that were 5′ end labelled with IRDye 700 or 800. Eight primer combinations were selected due to clarity and repeatability of bands (Table 2). Amplification products of duplexed selective PCR reactions were denatured and separated on 7 % acrylamide gels using a LI-COR 4200 DNA sequencer (LI-COR, Lincoln, NE, USA). One or more duplicate samples were routinely included in gel runs for quality control. The presence or absence of AFLP bands was scored manually using SAGA 2.0 MX software, and ambiguous bands were recorded as missing. Error rates (calculated as the number of mismatched genotypes divided by the number of bands) were 3–4 %.

**Table 2.** Combinations of EcoRI and MseI selective primers, and the total number of bands scored and per cent polymorphism (PLP ± SE) generated by each primer combination for *E. glaucus* populations

Primer pairs	# Bands	% PLP	% SE (PLP)
E-AAC/M-CAG	17	38.38	4.93
E-ACC/M-CAG	17	37.54	4.40
E-AAG/M-CGG	22	39.39	4.65
E-AGC/M-CGG	23	43.06	5.00
E-ACA/M-CAG	25	41.33	4.82
E-AGG/M-CAG	19	37.59	5.17
E-AAC/M-CGG	23	35.40	4.32
E-ACA/M-CGG	20	46.19	5.72
Mean	20.75	39.96	4.11

### Genetic analysis

We analysed AFLP marker data using methods previously employed for allotetraploid species, including band-based and allele-frequency metrics (e.g. Kang *et al.* 2005; Wagner *et al.* 2012). Band-based metrics compute distance-based measures of similarity within and among populations from the matrix of marker presence/absence data without inferring allele frequencies (Bonin *et al.* 2007). In contrast, allele-frequency methods estimate standard population genetic statistics; these methods were developed for diploid organisms and may not be valid for polyploid species. Allele-frequency methods were tested by Wagner *et al.* (2012) for an allotetraploid species, and estimated values correlated strongly with band-based metrics, since allotetraploid species likely undergo meiotic segregation within each parental genome similar to diploid species (Soltis and Soltis 1993).

### Genetic diversity

Using a band-based approach, we calculated the number and proportion of polymorphic bands (PPBs) within and among island and mainland sites by means of FAMD 1.2 software (Schlüter and Harris 2006). We also computed the number of fixed (or monomorphic) bands. The number of private and locally common bands (restricted to a limited area and found in ≤25–50 % of populations) was determined in GenAlEx 6.5 (Peakall and Smouse 2006). Differences in the number of polymorphic or fixed bands observed for island and mainland sites were compared using generalized linear models and a Poisson distribution in JMP 9 statistical software (SAS Institute Inc., Cary, NC, USA). The proportion of polymorphic loci (PLP) and expected heterozygosity (*H*_e_) were estimated for comparison to band-based metrics using a fragment-frequency approach in AFLP-SURV 1.0 (Vekemans 2002). This method assumes fixed homozygosity resulting from self-pollination and may overestimate marker frequencies for outcrossing taxa, but would meet expectations for *E. glaucus*. We subsequently compared band-based metrics with allele-frequency estimates using regression analysis. All files for genetic analyses were prepared using functions available in GenAlEx or the package AFLPdat (Ehrich 2006) for R software (R Development Core Team 2011).

### Genetic differentiation among populations and regions

We conducted a hierarchical analysis of molecular variance (AMOVA) in GenAlEx to partition genetic variation within and among populations nested in two regions: island and mainland. AMOVA components of variance include Φ_PT_, which is considered to be an analogue of *F*_ST_ (Wright 1951; Peakall and Smouse 2006). The significance of the different components of variance was tested with 9999 permutations. Values of Φ_PT_ were also calculated separately for populations within island or mainland regions. A Mantel test (10 000 permutations) was used to determine if there was an association between genetic distance measured as a matrix of linearized Φ_PT_ values, and log_10_ transformed geographic distances (Rousset 1997). The Mantel test was also conducted for the two subsets of island and mainland data. The indirect rate of gene flow (*N*_m_) was estimated following Wright (1951), and pairwise genetic distance (Φ_PT_) values were calculated and assayed for significance with 9999 permutations.

We compared AMOVA results for population genetic structure with allele-frequency estimates calculated in HICKORY software 1.1 (Holsinger *et al.* 2002; Holsinger and Lewis 2003). HICKORY employs a Bayesian approach to estimate *θ*^(II)^ (comparable to *F*_ST_ and Φ_PT_) using dominant markers, and does not assume Hardy–Weinberg equilibrium. We computed *θ*^(II)^ for three alternative models (full model, *f* = 0 model and *f*-free model) and selected a suitable model with the lowest deviance information criterion (DIC). Markov chain Monte Carlo (MCMC) parameters were set to default values (burn-in 50 000, sampling 250 000). To test for significant differences in population genetic structure among island and mainland locations, we ran posterior comparisons of *θ*^(II)^ values. If 95 % confidence intervals for the difference in paired samples included zero, we concluded that *θ*^(II)^ values were not significantly different.

To test the assumption that the 21 *E. glaucus* sites represented distinct populations, we used Bayesian clustering methods implemented in STRUCTURE 2.3.3 software to assign individuals to populations by employing the recessive alleles option for dominant markers (Pritchard *et al.* 2000; Falush *et al.* 2007). We ran 20 iterations of each *K* = 1–23 possible clusters using the default model that infers alpha and assumes admixture and correlated allele frequencies. Every run included a burn-in period of 150 000 MCMC cycles and 300 000 MCMC iterations (University of Oslo Bioportal; Kumar *et al.* 2009). The most likely number of clusters represented by the AFLP data was identified using the method described in Evanno *et al.* (2005) and implemented in HARVESTER software (Earl and vonHoldt 2012), which calculates Δ*K* as the second-order rate of change of the log probability of the data. CLUMPP 1.1.2 software (Jakobsson and Rosenberg 2007) aligned the 20 replicate runs and results were plotted with DISTRUCT 1.1 software (Rosenberg 2004).

## Results

### Genetic diversity

We scored clear and unambiguous AFLP bands as present or absent for each sampled individual. Of the 166 AFLP markers, 165 (99.4 %) were polymorphic for the full dataset. The proportion of missing data was calculated at 1.91 %. The average number of scorable bands generated by each AFLP primer combination was 20.75 (range of 17–25; Table 2). The number of polymorphic bands declined significantly for subsets of island or mainland data, reducing markers for analysis in some cases by more than half (Table 1). Mean genetic diversity for all samples was relatively high (PPB = 40.7 %), but varied considerably among populations (6–73 %). The lowest reported values for polymorphism were recorded for the population (SC4) on Santa Cruz Island with only 16 individuals, and may represent a recent founder event.

All values describing genetic diversity (PPB, PLP and *H*_e_) were strongly correlated (pairwise comparisons, *r* > 0.91, *P* < 0.0001), indicating concordance among band-based and allele-frequency metrics. The average expected heterozygosity (*H*_e_) within populations was low (0.1093) and varied among populations and locations (Table 1). The average expected heterozygosity within locations was greatest for the Catalina, VAFB and Sedgwick populations (0.152, 0.119 and 0.107, respectively) and declined for Santa Cruz (0.071) and Santa Rosa (0.097) Islands but was not significantly different among mainland and island locations. Comparisons of genetic diversity parameters between the two groups of mainland and island populations detected a significantly greater number of polymorphic bands among mainland populations (mean of 73 vs. 63 bands, respectively; *P* = 0.007). The mean number of fixed bands among populations did not differ significantly between mainland and island regions at the *P* = 0.05 level (67.8 vs. 62.2 bands; *P* = 0.101). Two private bands separated pooled island and mainland sites, and locally common bands were present among populations at each of the three island and two mainland locations (Table 1).

### Genetic differentiation among populations and regions

Most of the variation in the AFLP profiles reported here represented variation among populations within regions. Hierarchical AMOVA for the island and mainland data partitioned 37.1 % of the variation within populations, 55.8 % among populations within regions and 7.1 % between regions (Table 3). All values were significantly different from zero (*P* < 0.0001) and Φ_PT_ summed to 0.629. The average pairwise Φ_PT_ for all sites was 0.614 with a range from 0.159 (VAFB populations, V3 and V4) to 0.91 (Sedgwick and Santa Cruz populations). All pairwise comparisons for Φ_PT_ were significant **[see** **Supporting Information****]**. Population genetic structure declined within regions (Φ_PT_ = 0.422 among mainland and 0.400 among island populations) relative to structure calculated for all sites. The results of the Mantel test of linearized Φ_PT_ values, and log_10_ transformed geographic distances indicated no support for isolation by distance among all island and mainland sites (*P* = 0.175). This result was repeated when mainland sites were considered alone (*P* = 0.293). However, isolation by distance was detected among populations sampled on the three Channel Islands (matrix correlation coefficient = 0.153, *P* = 0.023). This result was limited to the groups of populations among islands and isolation by distance was not observed for populations within each island **[see****Supporting Information****]**. The value of *N*_m_, or the indirect rate of gene flow, was calculated as 0.147, indicating that the number of migrants each generation was significantly <1 and that genetic drift plays a role in population differentiation (Slatkin 1987).

**Table 3.** Analysis of molecular variance (AMOVA) results for *E. glaucus* populations located within and among the two (island and mainland) regions

Source of variation	d.f.	Sum of squares	Variance components	Variation (%)
Among regions	1	762.55	2.08	7.1
Among populations, within regions	19	336.02	16.42	55.8
Within populations	395	10.95	10.95	37.1

Using the Bayesian approach, two models of *θ*^(II)^ (full and *f* = 0) resulted in low DIC values, and we selected the full model with the expectation that the inbreeding coefficient (*f*) is >0 in this autogamous species (K. Holsinger, pers. comm.; Wilson *et al.* 2001). Under the full model, the value of *θ*^(II)^ for all 21 sites was 0.563, while values for the subsets of mainland or island sites were 0.557 and 0.556, respectively. Comparisons of posterior distributions detected no significant difference in population structure between island and mainland regions (difference in *θ*^(II)^ values: 0.001; confidence interval: −0.0379, 0.03932). Estimates of *θ*^(II)^ were not greater for populations located on islands relative to sampled populations on the mainland. Sedgwick and VAFB also did not differ significantly for estimates of *θ*^(II)^. However, Santa Catalina and Santa Rosa populations were significantly less differentiated than populations from Santa Cruz (difference in *θ*^(II)^ values: 0.189; confidence interval: 1349, 0.2445). Genetic structure was greater among sampled sites located on Santa Cruz Island.

**Figure 3.**
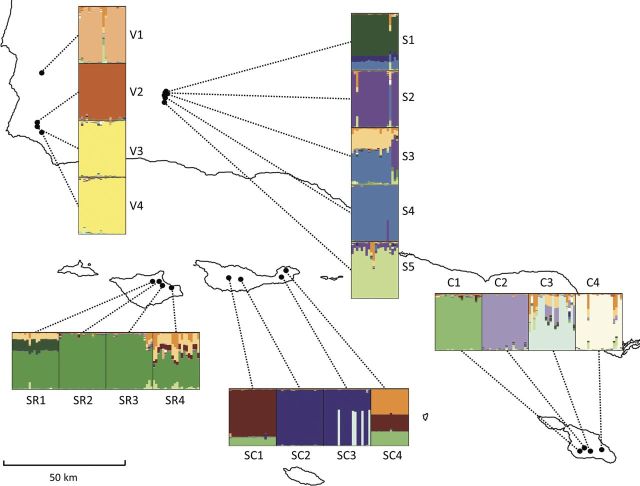
Estimated population structure for *K* = 16 in Bayesian analysis. Each of the study sites is represented by 21 segments and individuals within sites are designated by vertical columns. If individuals share the same pattern of genetic diversity, segments are homogeneous in colour.

Not all sampled populations were genetically distinct from one another. The highest likelihood of the number of genetic clusters represented in the AFLP dataset was consistently obtained with *K* = 16 (Fig. 3). In effect, the 21 collections represented 16 genetically distinct populations. Sites V3 and V4 corresponded to a single genetic cluster and these sites were located ∼3 km apart. Individuals among the four collection sites at Santa Rosa Island were also highly similar (2–7 km apart) despite their location in separate drainages on the northeastern shore of the island. Lastly, pairs of sites within Sedgwick and within Santa Cruz Island were overlapping. Altogether, evidence of admixture was low and plants derived from no more than five to seven of the original sites appeared to have patterns of diversity representative of dispersal and gene flow within islands (Fig. 3). Essentially, these individuals shared membership in more than one population. Most populations had fixed genetic differences that did not vary widely among individuals.

## Discussion

Strong levels of genetic differentiation observed in this study indicate that *E. glaucus* populations are highly self-pollinating and isolated at small distances. As a result, populations represent genetically homozygous lines and the distribution of diversity is heavily influenced by the breeding system (Schemske and Lande 1985). Similar patterns have been observed for previous studies of *E. glaucus* in North America, as well as among populations of other grass species worldwide (e.g. Jain and Allard 1960; Nevo *et al.* 1986; Knapp and Rice 1996). Predominantly self-pollinating species lead to different expectations for patterns of genetic diversity, and represent unique challenges for conservation that merit consideration when these species are targeted for reintroduction. In our study of *E. glaucus* among California Channel Island and mainland locations, we addressed questions regarding the levels and patterns of genetic diversity and differentiation at relatively isolated island sites, and their consequences for ecological restoration.

### Genetic diversity

Despite strong evidence for self-pollination, marker data indicated that considerable genetic variation is present in blue wildrye. Measures of the mean proportion of polymorphism within studied *E. glaucus* populations were ∼40 %. The distribution of genetic diversity may be the result of factors other than inbreeding, including the polyploid origin of *E. glaucus* as well as the potential, however small, for gene exchange among populations (Jain and Allard 1960). Genetic polymorphism present in alloploid grasses such as *E. glaucus* may be a consequence of segregation between homologous chromosomes within each parental genome, resulting in fixed heterozygosity in selfing lineages (Soltis and Soltis 1993). Additionally, self-pollination is rarely complete and gene exchange among lineages is possible through wind pollination in combination with seed dispersal among populations. Previous isozyme studies of *E. glaucus* growing in the northwestern United States and British Columbia also detected high levels of polymorphism at the species (77–80 %) and population level (22–31 %) (Knapp and Rice 1996; Ie 2000; Wilson *et al.* 2001), suggesting that high levels of genetic variation are maintained across this species' range.

Relative to the entire region sampled in this study, measures of genetic variation declined significantly when data were partitioned within island or mainland locations and particularly for individual populations, many of which were genetically homogeneous for AFLP markers. Mean expected heterozygosity (*H*_e_ = 0.109) for Channel Island and coastal California populations was low and corresponded to values recorded for selfing species with passively dispersed seeds (*H*_e_ = 0.097; Hamrick and Godt 1996), but varied among sampled sites. Populations located on Santa Cruz and Santa Rosa Islands had the lowest average levels of polymorphism while populations on Catalina Island were the most diverse. Levels of genetic diversity may vary among island and mainland locations, perhaps as a result of variation in the breeding system in addition to stochastic demographic and environmental factors (Schemske and Lande 1985).

Comparisons of the number of polymorphic bands between the two regions representing islands and the mainland indicated that genetic diversity was significantly higher for mainland populations relative to the Channel Islands. These data support predictions that island populations are genetically depauperate as a result of founder events, low rates of gene flow and subsequent genetic drift (Frankham 1997). Sampled populations for this study commonly represented fewer than 60 visible plants and locating populations on the islands was difficult, probably as a result of greater geographic isolation among *E. glaucus* populations on the islands. The consequences of relatively lower levels of diversity remain unclear, however. In many taxa, genetic diversity strongly correlates with population fitness and may be useful for identification of populations at risk of decline (Reed and Frankham 2003), but this pattern requires further investigation in self-pollinating species (Leimu *et al.* 2006). High levels of inbreeding can purge deleterious alleles so that selfing species are unlikely to experience inbreeding depression, and yet heterosis is commonly reported when gene exchange occurs (Jain and Allard 1960; Schemske and Lande 1985).

### Genetic differentiation among populations and regions

We discovered strong genetic differentiation in *E. glaucus* within and among island and mainland sites. Most of the variation was distributed among populations within regions and overall estimates of *F*_ST_ were very high (Φ_PT_ = 0.629, *θ*^(II)^ = 0.563) and similar to all prior studies of this species (Knapp and Rice 1996; Ie 2000; Wilson *et al.* 2001). These data point to the dominant role of the breeding system in shaping the population structure of *E. glaucus*. At the same time, relative levels of isolation may also affect patterns of genetic differentiation if the distance among populations further limits rare episodes of gene flow.

Tests for isolation-by-distance (IBD) were not significant for populations on the mainland and IBD was only apparent between islands. Two previous mainland studies of *E. glaucus* conducted at a larger scale also failed to detect IBD (Knapp and Rice 1996; Wilson *et al.* 2001), while low levels of IBD were detected for sites sampled throughout British Columbia (Ie 2000). Overall, genetic structure was not greater among Channel Island populations than among mainland populations and IBD was not observed among populations within each island.

Bayesian cluster analysis presented some evidence for admixture in *E. glaucus* as the result of gene flow, although this evidence was limited to few individuals and populations (e.g. SR4, Santa Rosa Island). Inference of genetic structure detected 16 genetically distinct populations among 21 collections, and confirmed strong population differentiation in *E. glaucus* among islands and the mainland. The average pairwise geographic distance among sites was ∼100 km. However, many sites within locations were only 2–3 km apart. We noted that the two closest collection sites (S1 and S2; 0.15 km apart) were strongly genetically differentiated. High levels of differentiation at small distances are common among self-pollinating grass species and represent challenges for their restoration in degraded ecosystems (Allard 1975).

### Implications for restoration

Evidence for lower levels of polymorphism among islands suggests that island populations of blue wildrye are genetically depauperate relative to the mainland. In these cases, the introduction of diverse seed sources in restoration has been proposed to increase population viability by reduction of inbreeding depression, and creation of opportunities for hybrid vigour (Broadhurst *et al.* 2008; Pekkala *et al.* 2012). Conversely, local seed collections are often proposed to avoid disruption of local adaptation and the incidence of outbreeding depression among hybrid progeny of remnant populations and introduced plants (Hufford and Mazer 2003). How do these contrasting views apply to restoration of *E. glaucus*?

If inbreeding depression is weak in self-fertilizing species (Schemske and Lande 1985), the preservation of genetic variation to conserve population viability should no longer be a large concern in reintroduction programmes. It does not follow, however, that the diversity of lines represented in seed mixes would also be of little concern. There is strong evidence for the maintenance of heterozygotes in selfing species, and heterozygote advantage has been proposed as a mechanism to improve population fitness when outcrossing is rare (Allard and Jain 1962). Ideally, when reintroducing species such as blue wildrye, the goal would be to maintain or even increase genetic variability among plants. In this case, the introduction of seeds representing large numbers of mixed lines would be supported (Broadhurst *et al.* 2008). However, non-local genotypes may represent plants poorly adapted to local environmental conditions.

High levels of genetic differentiation may be the product of both genetic drift and natural selection (Slatkin 1987), and evidence for local adaptation in *E. glaucus* has been detected in two reciprocal transplant studies (Hufford *et al.* 2008; Knapp and Rice 2011). In addition, other studies of blue wildrye detected high levels of fixed variation that were correlated with morphological variation among sites (e.g. Knapp and Rice 1996; Wilson *et al.* 2001; Erickson *et al.* 2004). In light of this evidence, population genetic divergence apparent in *E. glaucus* may reflect not only limited gene flow but also adaptation to heterogeneous environments. We noted that genetic structure declined when the full dataset was partitioned among islands and mainland locations (Φ_PT_ = 0.422 for the mainland and 0.400 for island populations relative to 0.629 among all sampled populations), and this scale corresponds to the scale of adaptive differentiation detected in field studies of this species. We consequently recommend the use of multiple seed collections specific to each Channel Island (for use at that island) to maintain diversity while avoiding long-distance introductions of non-local genotypes. Given low levels of pollen flow, these introductions are not likely to affect the viability of existing populations, and in rare cases where gene flow does occur, hybrid vigour among progeny would likely result in the formation of new selfing lines (Allard and Jain 1962; Nevo *et al.* 1986). Similar guidelines might apply to mainland locations, but would improve with further investigation of mainland sites.

We finish by noting that our recommendations are based on both evidence and speculation. It is unlikely that AFLP markers, which are presumed neutral, will correspond to adaptive differentiation in this species, and strategies for transfer of *E. glaucus* are derived from previous studies of adaptive variation as well as marker data (Kirk and Freeland 2011). Delineation of seed transfer zones, or regions within which translocation of plant materials is unlikely to result in the introduction of maladapted genotypes, is currently the best method to predict adaptive divergence and select suitable germplasm for restoration (e.g. Johnson *et al.* 2004). Yet, data for adaptive differentiation at the scale of contemporary grassland restoration are scarce. Additional research (e.g. Hufford *et al.* 2008) to examine the scale of adaptive divergence among populations in sensitive regions would benefit restoration of temperate grasslands. In the interim, marker data can assist with the selection of germplasm to minimize the risk of introductions and yet maintain high levels of genetic diversity. Restoration sites, including large-scale seeding efforts under way across the western United States (Burton and Burton 2002), will serve as the proving ground for marker predictions of short- and long-term population viability in contemporary and changing environments.

## Sources of Funding

This research was supported by the National Parks Ecological Research Fellowship Program, a programme funded by the National Park Foundation through a generous grant from the Andrew W. Mellon foundation.

## Contributions by the Authors

K.M.H., S.J.M. and S.A.H. conceived and designed the study. K.M.H. and S.J.M. conducted field collections. K.M.H. conducted laboratory and data analyses and wrote the manuscript with the help of S.J.M. and S.A.H.

## Conflicts of Interest Statement

None declared.

## Supporting Information

The following files are available in the online version of this article –

**Table S1.** Pairwise genetic distance (Φ_PT_) values among the 21 sampled *E. glaucus* populations.

**Figure S1*.*** Relationship between linearized Φ_PT_ values and log_10_ values for geographic distance among populations of *E. glaucus* distributed on Santa Rosa, Santa Cruz and Catalina Islands off the coast of mainland California. Axes are oriented to highlight clusters of points representing each island.

Additional Information
