# Supplementary material for: Genetic variation among mainland and island populations of a native perennial grass used in restoration
Source: AoB Plants. 2013 Dec 18;6:plt055. doi: 10.1093/aobpla/plt055 (PMC3966692; doi:10.1093/aobpla/plt055)
Supplement: Additional Information [file supp_6_plt055_index.html]

Genetic variation among mainland and island populations of a native perennial grass used in restoration — Additional Information 

# Genetic variation among mainland and island populations of a native perennial grass used in restoration

## Additional Information

Additional Information

**Files in this Data Supplement:**

- Additional Information Figure 1 - tit file
- Additional Information Table 1 - xlsx file
