# Supplementary figures and images for: Genetic variation among mainland and island populations of a native perennial grass used in restoration
Source: AoB Plants. 2013 Dec 18;6:plt055. doi: 10.1093/aobpla/plt055 (PMC3966692; doi:10.1093/aobpla/plt055)

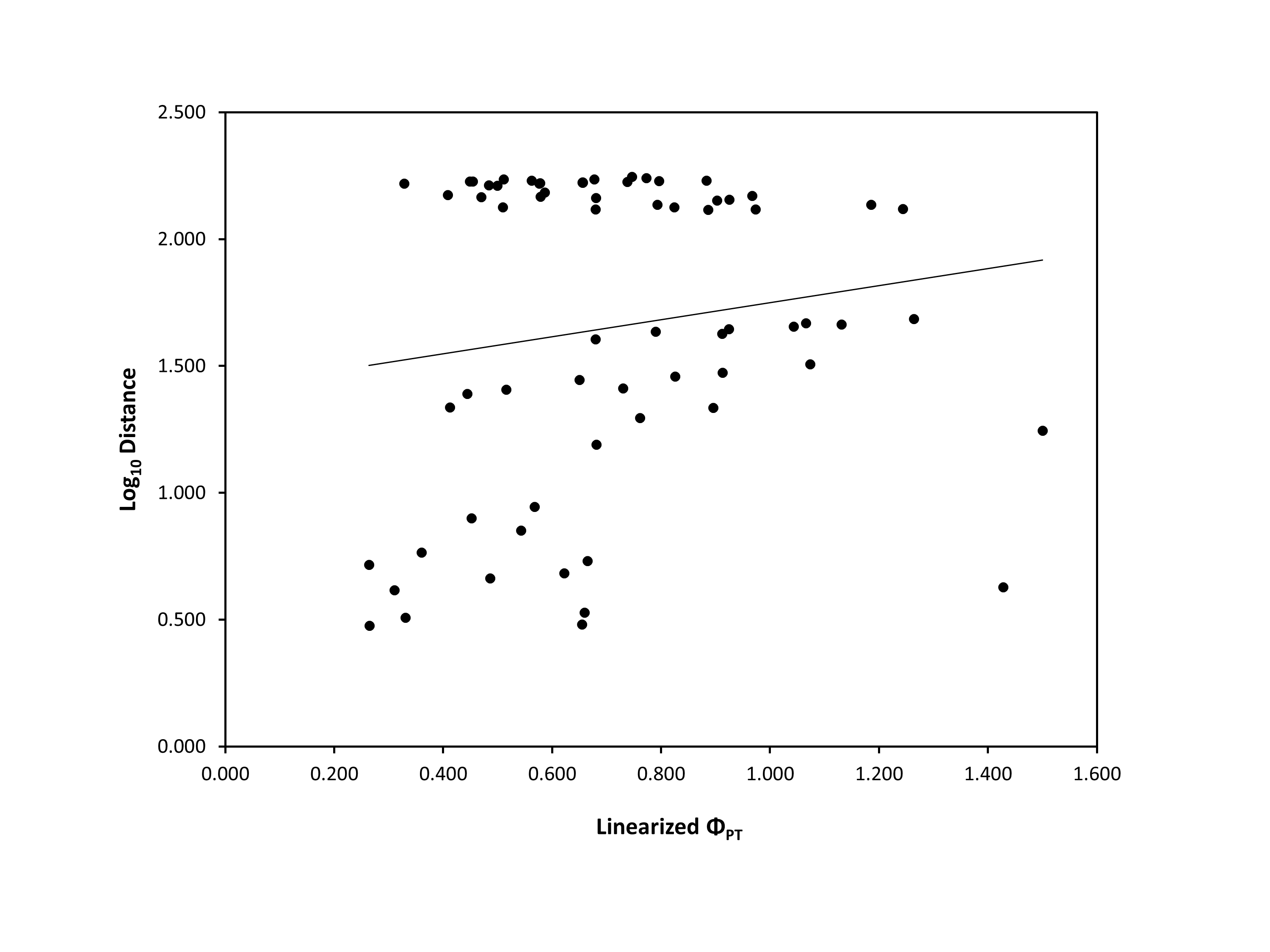

Supplement: Additional Information [file supp_plt055_plt055supp_fig.tif]
